# Supplementary material for: Antibody signatures elicited by potent and subpotent whole-cell pertussis vaccines in mice
Source: Microbiol Spectr. 2025 Mar 25;13(5):e03253-24. doi: 10.1128/spectrum.03253-24 (PMC12054088; doi:10.1128/spectrum.03253-24)
Supplement: Supplemental figures — Figures S1 to S3. [file spectrum.03253-24-s0001.pdf]

Tables S1-S9. Provided as Excel File

Reactive *B. pertussis* antigens recognized by wP immune sera at all stress temperatures relative to sham vaccinated animals

Figure S1

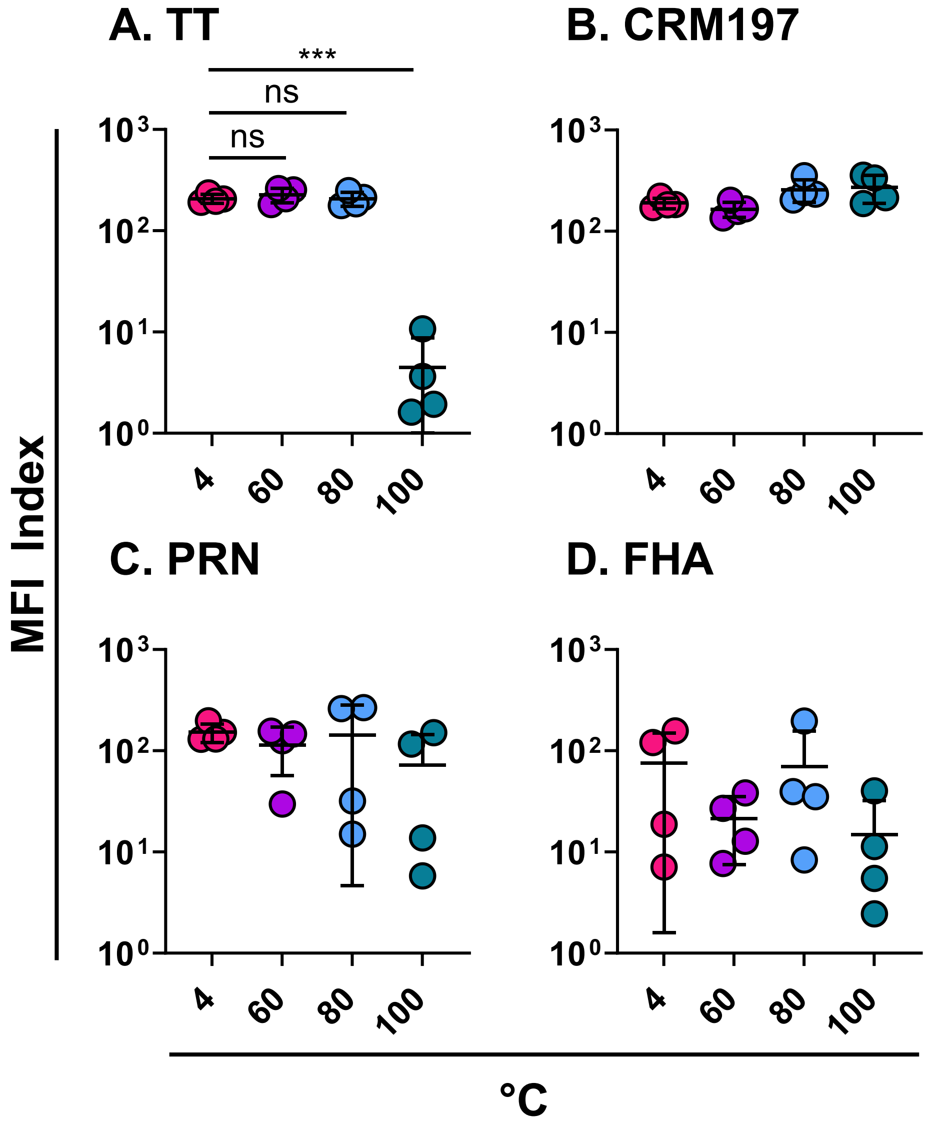

Figure S1. Antigen-specific responses to DTwP$_{4, 60, 80, 100}$. Mice (n=4 per group) were intraperitoneally vaccinated with DTwP stressed at different temperatures on days 0 and 21 with 1.2 OU/mouse. Sera was collected on day 30 pre-challenge and was analyzed by MIA to determine antigen-specific titers.

Figure S2

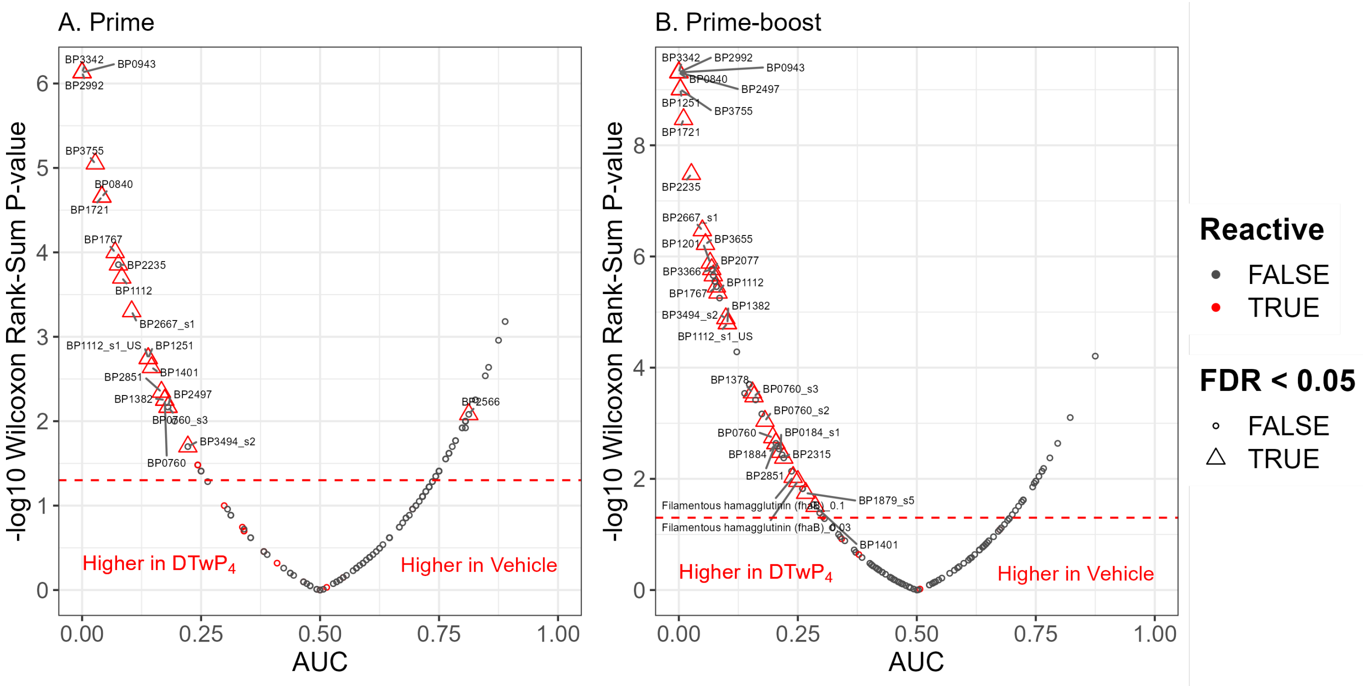

Figure S2. Antigens specifically associated with DTwP$_4$ vaccination. Serum samples from BALB/c mice (n=51) vaccinated with DTwP$_4$ on day 0 (prime) or day 0 and 21 (boost); alongside sham vaccinated mice (n=44) were subjected to the *B. pertussis* proteome array. The identified immunoreactive IVTT *B. pertussis* antigens were rank ordered based on AUC and statistical significance.

Figure S3

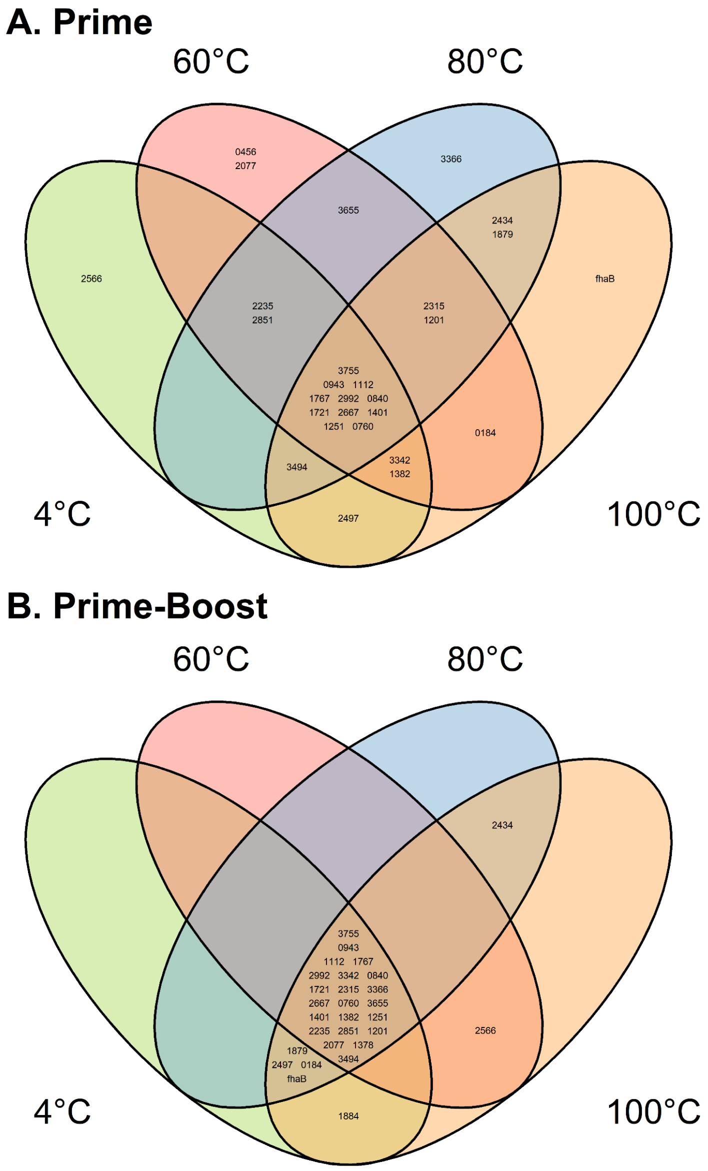

Figure S3. Venn diagram showing overlaps between differential reactive antigens across all stress temperatures. To identify unique serological signatures, differential reactive antigens in the serum samples from prime (d0) and boost (d0 and d30) $DTwP_{4,\ 60,\ 80\ ,100}$ vaccinated animals were compared for overlaps at all stress temperatures.
